# The influence of duration on pain stress, oxidative stress, and total antioxidant power status in female dogs undergoing ovariohysterectomy

**DOI:** 10.14202/vetworld.2020.160-164

**Published:** 2020-01-23

**Authors:** Kanissarinn Sakundech, Chayanon Chompoosan, Pongsatorn Tuchpramuk, Thongchai Boonsorn, Worapol Aengwanich

**Affiliations:** Stress and Oxidative Stress in Animal Research Unit, Faculty of Veterinary Sciences, Mahasarakham University, Mahasarakham 44000, Thailand

**Keywords:** female dog, ovariohysterectomy, oxidative stress, pain stress, total antioxidant power

## Abstract

**Background and Aim:**

Ovariohysterectomy (OHE) is a common procedure for sterilization of female dogs. However, knowledge of changes in pain stress, oxidative stress, and total antioxidant power status before, during, and after OHE is limited. The objective of this experiment was to study the effect of duration on pain stress, oxidative stress, and total antioxidant power status in female dogs undergoing OHE.

**Materials and Methods:**

Seven female dogs were sterilized using the OHE method. Pain scores, hematological changes, and biochemical markers were investigated during pre-operative, 3 h after starting OHE, and on days 3, 7, 10, and 14 of an experimental period. Data were analyzed using one-way analysis of variance.

**Results:**

At 3 days after OHE, pain score was higher than on days 7-14 of the experimental period; percentage of neutrophil, 3 h after starting OHE, was higher than during pre-operative and on days 3-14 of the experimental period; percentage of lymphocyte on days 10-14 was lower than during pre-operative, 3 h after starting OHE, and on days 3-7 of the experimental period; neutrophil/lymphocyte ratio, 3 h after starting OHE, was higher than during pre-operative and on days 3-14 of the experimental period; plasma malondialdehyde on day 3 was higher than during pre-operative, 3 h after starting OHE, and on days 3-14 of the experimental period; and total antioxidant power on day 14 was higher than during pre-operative, 3 h after starting OHE, and on days 3-10 of the experimental period, respectively.

**Conclusion:**

This experiment indicated that OHE caused pain stress, oxidative stress, and reduction of total antioxidant power in female dogs. Finally, female dogs needed antioxidant for 7 days after OHE.

## Introduction

Stray dog overpopulation and control of its menace to human beings and domestic animals are a matter of socioeconomic importance in developing countries. The relationship between a community and its dogs is not always entirely positive as stray dogs cause road accidents, barking and fighting, biting children, killing livestock, and uncontrolled fecal contamination [1]. Potential benefits of sterilization are population and disease control; inhibition of diseases related to reproductive tract [2], treat diseases associated with the reproductive system, such as mammary neoplasia, alleviation of the risk of pyometra, and estrus attraction of male dogs resulting in inconvenience to the owner [1].

Ovariohysterectomy (OHE) is a common procedure for sterilization of female dogs [1,3,4]. The operation removes both ovaries, the uterine horns and the body of the uterus [2]. Surgical stress occurs before, during, and after an operative procedure. It arises from psychological stress, tissue injury, and alterations in circulation, anesthetic agents, and post-operative complications including sepsis. The surgical stress response involves the stimulation of the sympathoadrenal medullary and the hypothalamic–pituitary–adrenal axis. Their activation causes endocrine and immunomodulatory changes after trauma. The degree of physiological response is proportional to the magnitude of injury with increased demands on organ function. Response to surgical stress is a compensatory mechanism that prevents secondary damage and increases the availability of substrates required by essential organs and healing tissues. However, if the stress response is prolonged, it results in longer hospitalization. Therefore, alleviating prolonged pain and stress in surgical patients is important for animal health [4]. The stress response is a physiological response to trauma or surgery and is generally considered to be proportional to the degree of surgical trauma [5]. Hematological biomarkers [2,6] such as the percentage of neutrophil, lymphocyte [7], and neutrophil/lymphocyte [8] are measured commonly for the evaluation of stress response [2,7,8]. During cellular respiration, oxygen, the key element required to produce energy for the cellular metabolism and oxidation of organic compounds, is consumed and reduced, generating a series of highly reactive chemical substances called reactive oxygen species (ROS), also known as free radicals [9]. Oxidative stress is a phenomenon caused by an imbalance between production and accumulation of ROS [10]. In general, the trauma of a surgical procedure may contribute to oxidative stress [11]. The result of the reactions of ROS with biomolecules is the formation of substances that can be used as markers of oxidative damage [9] such as malondialdehyde (MDA) [11]. To counteract the harmful effects taking place in the cell, the system has evolved with some strategies such as prevention of damage, repair mechanism to alleviate the oxidative damages, physical protection mechanism against damage, and the final most important is the antioxidant defense mechanisms. Based on the oxidative stress-related free radical theory, the antioxidants are the first line of choice to take care of stress. Endogenous antioxidant defenses include a network of compartmentalized antioxidant enzymatic and non-enzymatic molecules that are usually distributed within the cytoplasm and various cell organelles [12]. Catalase is an important endogenous antioxidant enzyme that catalyzes H_2_O_2_ detoxification [13]. In normal conditions, it is the most adaptive antioxidant enzyme to play a significant role in cell defense against oxidative damage in the presence of oxidative stress [14]. Total antioxidant power considers the cumulative effect of all antioxidants present in blood and body fluids. It is considered as a useful indicator of the body’s antioxidant status to counteract the oxidative damage due to ROS [15]. Many previous studies have found that OHE caused pain stress [16] and oxidative stress [11].

However, knowledge of changing of pain stress, oxidative stress, and total antioxidant power status before, during, and after OHE is limited. Therefore, the objective of this experiment was to study the effect of duration on pain score, hematological changes, oxidative stress, and total antioxidant power before, during, and after OHE in female dogs.

## Materials and Methods

### Ethical approval and informed consent

This study was approved by the Institution’s Ethics Committee on Animal Experimentation of Mahasarakham University (license number: 011/2561). All procedures were performed with the owner’s consent.

### Experimental design

The experimental design of this study was completely randomized design with six treatments (times were treatment) as follows: (treatment 1) During pre-operative, (treatment 2) 3 h after starting OHE, (treatment 3) day 3, (treatment 4) day 7, (treatment 5) day 10, and (treatment 6) day 14 of experimental period, respectively. During the experimental period, dogs received feed and water *ad libitum*.

### Methods

#### Animals

The study was carried out on seven clinically healthy mixed-breed domestic female dogs (aged, 1-3 years) with a mean (standard deviation [SD]) weight of 12±5 kg. Animals were evaluated according to their history, absence of any previous illness, and the absence of drug or dietary supplement. General clinical examinations were performed and recorded. Blood samples of experimental groups were taken from a cephalic vein and only occasionally from a saphenous vein for hematological (complete blood count and platelets) and biochemical (creatinine, alanine aminotransferase, and total protein) analysis. Blood parasites of all dogs were determined. All animals were submitted to conventional vaccination and deworming protocols. Before starting the experiment, dogs were kept at an experimental unit for at least 7 days. Dogs were placed under a fasting state 6 h before surgery.

#### Sedation and anesthesia

Dogs were sedated with xylazine (1.1 mg/kg body weight [BW]) by subcutaneous injection. After the onset of sedation, a 22 gauge intravenous (IV) catheter was placed in the cephalic vein. Normal saline (0.9% NSS, 10 mL/kg BW/h) was administered through IV catheter with cefazolin sodium (22 mg/kg BW). All dogs received tramadol hydrochloride (4 mg/kg BW) by subcutaneous injection. After administering xylazine for 15 min, animals were anesthetized using thiopental sodium (10-25 mg/kg BW) through an IV catheter with 0.9% NSS until intubation. The anesthetized state was maintained using thiopental sodium throughout the surgery. During anesthesia, vital signs were monitored at 5 min intervals. The level of anesthesia was evaluated by reflex examination.

#### Surgery

OHE was performed according to the technique described by Tobias [17].

#### Post-operative care

After finishing the surgery, animals were immediately given yohimbine hydrochloride (0.11 mg/kg BW) through an IV catheter with 0.9% NSS. Tramadol hydrochloride (4 mg/kg BW) was administered by subcutaneous injection for 6 h after the initial application and then every 12 h for 5 days. Cephalexin sodium (20-30 mg/kg BW) was administered peroral 2 times after meals for 14 days.

### Determination of pain score, hematological parameters, and laboratory analysis

#### Pain score

Pain scores were assessed using the Glasgow Composite Measure Pain Scale [18]. This assessment was performed after dog recovery, on days 3, 7, 10, and 14 of the experimental period.

#### Hematological and biochemical analysis

Blood samples were collected pre-operative, after starting OHE for 3 h, on days 3, 7, 10, and 14 of the experimental period from the saphenous vein using a butterfly needle into vacuum ethylenediaminetetraacetic acid (EDTA) and heparin tube for hematological and biochemical analysis, respectively, and then centrifuged at 2500 rpm for 5 min. The obtained heparinized plasma was frozen in cryotubes and stored at −20°C before biochemical analysis. Blood cell differential count and calculation

Blood samples with added EDTA as an anticoagulant were placed immediately on ice and were transferred to the laboratory. Blood films were prepared, fixed with methanol, and stained with Giemsa-Wright solution and then used for a white blood cell differential count. The neutrophil/lymphocyte ratio was calculated.

Plasma MDA

MDA in plasma was investigated using the procedure as followed: 0.01 mL of sample was assayed by the addition of 3 mL (0.05 mol/L) of HCl and 1 mL (0.67%) of thiobarbituric acid. Cocktails were heated for 30 min at 100°C, cooled with running tap water, and then 4 mL of n-butyl alcohol was added. The mixture was shaken in a vortex mixer and centrifuged at 3000 rpm (1008× *g*) for 10 min. The absorbance at 532 nm was compared with that of 1,1,3,3-tetramethoxypropane standards.

Plasma total antioxidant power

Plasma total antioxidant power was evaluated using the ferric reducing ability of plasma (FRAP) assay, the procedure as followed: 300 mmol/L of acetate buffer (pH 3.6), 10 mmol/L of 2,4,6-tri-pyridyl-s-triazine in 40 mmol/L of HCl, and 20 mmol/L of FeCl_3_.6H_2_O were prepared. Twenty milliliters of acetate buffer, 2.5 mL of 2,4,6-tri-pyridyl-s-triazine, and 2.5 mL of FeCl_3_.6H_2_O yielded the working FRAP reagent, and then, 10 mL of plasma, 10 mL of deionized distilled water, and working FRAP reagent were mixed. After exactly 6 min at room temperature, absorbance at 593 nm was read against reagent blank. Fe (II) at 100-1000 mmol/L was used as the standard.

Plasma catalase activity

Catalase activity was made by spectrophotometric measurement of decreasing H_2_O_2_ quantity, the procedure as followed: 0.05 M potassium phosphate, pH 7.0, and 0.059 M hydrogen peroxide (30%) in 0.05 M potassium phosphate, pH 7.0. The spectrophotometer was adjusted to 240 nm at room temperature. A pipette was used to transfer 1.9 mL of reagent grade water and 1.0 mL of 0.059 M hydrogen peroxide to cuvettes. These were incubated in the spectrophotometer for 4-5 min to achieve temperature equilibration. After equilibration, 0.1 mL of the sample was added and a decrease in absorbance at 240 nm for 2-3 min was recorded. Catalase enzyme was used as a standard.

### Statistical analysis

Data were analyzed using one-way analysis of variance. Means were separated by Duncan’s multiple range tests. All results were expressed as the mean±SD. The level of significance was determined at p<0.05.

## Results and Discussion

A study was conducted on seven female dogs on the effect of duration on pain stress, oxidative stress, and total antioxidant power status before, during, and after OHE surgery. Assessments were made during pre-operative, 3 h after starting OHE surgery, and on days 3, 7, 10, and 14 of an experimental period. The results revealed the following information: Pain score, 3 days after OHE, was significantly higher than on days 7, 10, and 14 of the experimental period (p<0.05); percentage of neutrophil, 3 h after starting OHE, was significantly higher than during pre-operative and on days 3, 7, 10, and 14 of the experimental period (p<0.05); the percentage of lymphocyte on days 10 and 14 was significantly lower than during pre-operative, 3 h after starting OHE, and on days 3 and 7 of the experimental period (p<0.05); neutrophil/lymphocyte ratio, 3 h after starting OHE, was significantly higher than during pre-operative and on days 3, 7, 10, and 14 of the experimental period (p<0.05); plasma MDA on day 3 was significantly higher than during pre-operative, after starting OHE for 3 h, and on days 3, 7, 10, and 14 of the experimental period (p<0.05); and total antioxidant power on day 14 was significantly higher than during pre-operative, 3 h after starting OHE, and on days 10, 7, and 3 of the experimental period (p<0.05), respectively. The catalase throughout the experimental period was not significantly different (p>0.05) (Table-1).

**Table-1:** Effect of duration on pain stress, hematological changes, and biochemical values before, during, and after OHE in female dogs.

Parameters	Pre-operative	After starting OHE for 3 h	Day 3 after OHE	Day 7 after OHE	Day 10 after OHE	Day 14 after OHE	SEM
BW (kg)	12.71±3.67	12.71±3.67	12.56±3.66	12.45±3.60	12.63±3.52	12.46±3.42	1.36
Clinical sign							
Pain score	-	-	1.43±1.81a	0.14±0.38b	0.00±0.00b	0.00±0.00b	0.35
Hematology							
Neutrophil (%)	83.14±1.68b	90.86±2.91a	75.14±9.67c	76.57±7.76bc	62.29±8.10d	60.00±6.66d	2.56
Lymphocyte (%)	9.57±1.81bc	5.50±1.52c	14.86±7.43b	15.86±8.21b	25.43±11.89a	32.57±8.00a	2.86
N/L ratio	8.96±1.76b	17.38±4.33a	7.78±6.74b	6.18±3.42bc	2.98±1.41c	1.97±0.64c	1.39
Biochemistry							
MDA (μM)	21.97±3.00b	21.00±2.21b	26.83±3.71a	22.18±1.60b	19.95±3.35b	19.91±3.45b	1.12
FRAP (mM)	1.07±0.14bc	1.01±0.17c	1.06±0.08bc	1.16±0.12ab	1.19±0.12ab	1.27±0.07a	0.00
Catalase activity (units/mg protein)	211.57±91.55	213.00±108.93	236.33±92.45	247.29±111.78	264.43±149.38	368.71±230.86	53.18

BW=Body weight, N/L ratio=Neutrophil/lymphocyte ratio, MDA=Malondialdehyde, FRAP=Ferric reducing ability of plasma, OHE=Ovariohysterectomy, SEM=Standard error of the mean. Within rows, means with no common superscript differ significantly (p<0.05)

Post-operative pain is acute and associated with actual or potential tissue damage [19]. It is classified as nociceptive pain occurring when peripheral neural receptors are activated by noxious stimuli. This pain results from activation of the immune system and inflammation in response to injury [20]. Due to the lack of verbal communication, the level of post-operative pain in dogs is difficult to assess. Therefore, the assessment of pain relies on temperament, vocalization, posture, activity level, locomotion, reaction to palpation, and other behavioral changes [19]. In the present study, we found that 3 days after OHE, pain score was higher than on days 7, 10, and 14 of the experimental period. This showed that on day 3 after OHE, the wound from tissue injury was under inflammation and not yet healed. Therefore, dogs were under pain and showed high pain scores. The result followed the study of Quarterone *et al*. [21]. They found that OHE produces high expression of pain because the surgical procedure caused high tissue injury.

Evaluating the hematological characteristics is an important tool that can be used as an effective and sensitive index to monitor physiological and pathological changes in animals. The analysis of blood indices has proven to be a valuable approach for analyzing the health status of animals as these indices provide reliable information on health and stress status. It is well documented that OHE inflicts pain and stress as a result of tissue trauma, organ manipulation, and inflammation [22]. Hancock *et al*. [23] reported a significantly higher peak plasma cortisol level in dogs 2 h after OHE. In response to the stress hormone, neutrophils shift from the marginated to the circulating neutrophil pool, but this neutrophilia might also be enhanced by the release of neutrophils from the bone marrow storage pool and the decreased emigration of neutrophils to the tissues [8]. Besides, Arunkumar *et al*. [1] observed neutrophilia and lymphocytopenia in ovariohysterectomized dogs. Like the present study, the percentage of neutrophil 3 h after starting OHE was higher than pre-operative and on days 3, 7, 10, and 14 of the experimental period. On the other hand, the percentage of lymphocyte, 3 h after starting OHE, was lower than pre-operative and on days 3, 7, 10, and 14 of the experimental period. Therefore, the neutrophil/lymphocyte ratio, 3 h after starting OHE, increased. MDA level is a very useful indicator of lipid peroxidation occurring under oxidative stress. The plasma MDA concentration is frequently used as a biomarker for overall lipid peroxidation [24]. In the study, plasma MDA of female dogs on day 3 was higher than pre-operative, 3 h after starting OHE, and on days 7, 10, and 14 of the experimental period. This phenomenon showed that dogs on day 3 of the experimental period were under oxidative stress. Finally, the total antioxidant power of female dogs on day 14 was higher than during pre-operative, 3 h after starting OHE, and on days 10, 7, and 3 of the experimental period, respectively. This experiment showed that on day 14 of the experimental period, the total antioxidant power of female dogs was higher than during pre-operative because dogs were under fasting and water deprivation before an operation. These processes may be caused by dogs under oxidative stress and a decrease of total antioxidant power when compared with day 14 of the experimental period. The reduction of total antioxidant power during pre-operative, 3 h after starting OHE, and on days 10, 7, and 3 of the experimental period followed the level of MDA that was highest on day 3 of the experimental period. This phenomenon indicated that after OHE, dogs were under oxidative stress which caused a reduction of total antioxidant power. Therefore, they needed antioxidants to protect their bodies from free radicals between faced to oxidative stress conditions after OHE for 7 days.

## Conclusion

OHE caused female dogs pain stress, oxidative stress, and reduction of total antioxidant power. However, dogs could adapt to pain stress within 7 days after OHE. Dogs need an antioxidant for 7 days after OHE.

## Authors’ Contributions

KS and WA designed the experiment and collected data. CC and PT partially collected samples. KS and TB conducted laboratory examinations. WA scientifically analyzed the result, drafted, and revised the manuscript. All authors read and approved the final manuscript.
